# Associations of visit-to-visit variabilities and trajectories of serum lipids with the future probability of type 2 diabetes mellitus

**DOI:** 10.1186/s12944-021-01592-9

**Published:** 2021-11-27

**Authors:** Qian Sun, Jingchao Liu, Lei Wu, Yue Sun, Jianrong Jin, Sudan Wang, Jing Wu, Yang Jing, Hui Zhou, Chen Dong

**Affiliations:** 1 Department of Epidemiology and Statistics, School of Public Health, Jiangsu Key Laboratory and Translational Medicine for Geriatric Disease, Medical College of Soochow University, Soochow, Jiangsu China; 2 Suzhou Wuzhong Centers for Disease Control and Prevention, Soochow, China; 3 Suzhou Industrial Park Centers for Disease Control and Prevention, Soochow, China

**Keywords:** Type 2 diabetes mellitus, Serum lipids, Visit-to-visit variability, Trajectory, Association

## Abstract

**Background:**

Serum lipid abnormalities are generally considered as a major risk factor for type 2 diabetes mellitus (T2DM). However, evidence for the effect of long-term serum lipid fluctuations on future T2DM probability remains limited.

**Methods:**

A total of 4475 nondiabetic participants who underwent annual health examinations between 2010 and 2013 were followed for the subsequent 5-year risk of T2DM. The Cox proportional hazards model was performed to evaluate the associations of visit-to-visit variabilities and trajectories of triglycerides (TG), total cholesterol (TC), high-density lipoprotein cholesterol (HDL-c) and low-density lipoprotein cholesterol (LDL-c) with T2DM probability.

**Results:**

During the five-year follow-up, 223 newly developed T2DM cases were identified. Compared with the “Low” TG trajectory, “Moderate” and “Moderate-High” TG trajectories were significantly associated with T2DM incidence, with adjusted hazard ratios (HRs) and 95 % confidence intervals (CIs) of 1.51 (1.12-2.03) and 2.55 (1.62-4.03), respectively. Additionally, participants in the third and fourth quartiles of TG/standard deviation (SD) were associated with increased T2DM probability when compared with those in the lowest quartile. After excluding individuals with prediabetes, participants with “Moderate-High” TG trajectory still had a 2.43-fold greater risk of T2DM compared with those with “Low” TG trajectory (95 % CI: 1.28-4.63). In addition, compared with participants in “Low” HDL-c trajectory, the future T2DM probability was significantly reduced in those with “Moderate” and “High” HDL-c trajectories, with HR (95 % CI) of 0.52 (0.37-0.72) and 0.38 (0.18-0.80), respectively. After excluding individuals with prediabetes, the “Moderate” HDL-c trajectory remained associated with decreased T2DM probability when compared with “Low” HDL-c trajectory (HR: 0.55, 95 % CI: 0.35-0.88). However, the incidence of T2DM was not associated with the long-term fluctuations of TC and LDL-c.

**Conclusions:**

Long-term visit-to-visit variability of TG, and the change trajectories of TG and HDL-c were significantly associated with future T2DM probability. Moreover, these associations were not affected after excluding individuals with prediabetes.

**Supplementary Information:**

The online version contains supplementary material available at 10.1186/s12944-021-01592-9.

## Introduction

Worldwide, type 2 diabetes mellitus (T2DM) is widely recognized as a considerable cause of morbidity and mortality. In China, the latest statistical data showed that prediabetes is prevalent in 35.2 % of people older than 20-year-old. Moreover, in 2013, 98.4 million adults aged 20-79 years had diabetes, and in 2020 this number increased to 129.8 million. Of these cases, 90 %-95 % were diagnosed with T2DM [1–3]. The escalating rates of T2DM signify a serious disease burden on the total health care system as well as at the individual levels [4, 5]. Thus, it is still vital to explore more T2DM risk factors and to develop more efficient preventive measures.

Lipid abnormalities, including an elevated blood concentration of triglyceride (TG), total cholesterol (TC), low-density lipoprotein cholesterol (LDL-c), and reduced level of high-density lipoprotein cholesterol (HDL-c), are generally considered as important risk factors for T2DM [6–8]. However, the abnormalities of serum lipid species exhibited in T2DM patients are different. For example, a Nigerian study reported that dyslipidemia was present in 69.3 % of T2DM patients. Among them, the prevalence of mixed dyslipidemia of high LDL-c and high TG was 41.0 %, while only 2.5 % of T2DM cases were determined with low HDL-c and high TG [9].

Based on the previous findings, it is reasonable to hypothesize that long-term changes in serum lipids of different species should have different effects on T2DM development. Therefore, this study was conducted to explore the visit-to-visit variabilities and trajectories of TC, TG, HDL-c and LDL-c between 2010 and 2013, and to test their associations with the subsequent 5-year T2DM probability.

## Methods

### Population

All participants were recruited from the study of “The prevention of MS and multi-metabolic disorders in Jiangsu province of China” (PMMJS) [10–12]. Briefly, 5769 individuals (aged 30-65 years old) who underwent an annual physical examination from 01/01/2010 to 12/31/2010, were asked to join in this study. To characterize the serum lipid fluctuations between 2010 and 2013 and to test their effects on the subsequent 5-year risk of T2DM, individuals who met the following criteria were excluded: (a) history of diabetes (including type 1 diabetes and T2DM) before 2013; (b) high glycosylated hemoglobin A1c (HbA1c) (≥ 6.5 %) before 2013; (c) high fasting plasma glucose (FPG) (≥7.0 mmol/L) before 2013; (d) participants who had serious diseases of the lung, kidney, heart and tumor before 2013.

A face-to-face survey was conducted with every participant by trained interviewers for lifestyle evaluation including age, gender, smoking (≥ 1 cigarette/day consumption over 6 months), drinking (≥ 1 time/week alcohol consumption over 6 months) and medical history. Anthropometric measurements were carried out with experienced physicians. The formula of weight/height^2^ (kg/m^2^) was used for body mass index (BMI) calculation. Hypertension was defined according to the criteria described previously [13].

Serum TC, TG, HDL-c and LDL-c concentrations were analyzed in 2010, 2011, 2012 and 2013, respectively. Plasma levels of HbA1c and FPG were monitored annually, and newly diagnosed T2DM cases were consecutively enrolled from 2014 to 2018. After excluding the subjects who were lost to follow-up including death and those with missing data, the current study ultimately included 4475 adults (3125 males and 1350 females) for the analysis.

### Laboratory measurement

Blood samples were drawn from each participant after fasting for at least 8 h. The serum and plasma samples were separated immediately. Serum TC, TG, HDL-c and LDL-c concentrations, and plasma FPG and HbA1c concentrations were analyzed using a conventional automated blood analyzer (Olypums AU640, Japan).

### Ascertainment of T2DM and prediabetes

An incident case of T2DM was diagnosed as the following criteria [14]: (a) FPG ≥ 7.0 mmol/L or HbA1c ≥ 6.5 %; (b) 2-hour oral glucose tolerance test ≥ 11.1 mmol/L; (c) Use of antidiabetic drugs; (d) Self-report of T2DM diagnosed by physician. All cases that were diagnosed as type 1 diabetes, secondary diabetes, or others types of diabetes were excluded.

In this study, prediabetes was determined according to the following criteria [14]: (a) the level of 2-hour oral glucose tolerance between 7.8 mmol/L and 11.0 mmol/L and/or (b) the level of FPG between 6.1 mmol/L and 6.9 mmol/L.

### Statistical analysis

Data were described as mean (standard deviation, SD) and percentage for continuous variables and categorical variables, respectively. The Student’s *t*-test and the Pearson’s χ^2^ test were respectively applied for normally distributed continuous variables and categorical variables analysis. The coefficient of variation (CV) and SD were used as indices of visit-to-visit variability to obtain more details. The trajectories of serum lipids from 2010 to 2013 were determined using latent mixture modeling within the PROC TRAJ procedure in SAS macro. In brief, the models of serum lipid trajectories were firstly estimated using Censored normal distributions with imputed numbers (two, three, four, five, and so on) and shapes (linear, quadratic, cubic, etc.). Next, Bayesian information criterion was applied for the evaluation of the model fit, and the model with the smallest negative number was assessed as the best-fit model. Then, the model with different functional forms was compared by examining the mean posterior probability of group membership (> 0.70 is recommended conventionally) [15, 16]. The latent class trajectory analysis automatically divided the population into classes, such that participants in the same class tended to be with similar trajectories. The prospective associations of incident T2DM with the mean levels, visit-to-visit variabilities and trajectories of serum lipids were evaluated using hazard ratios (HRs) with 95 % confidence intervals (CIs) based on the Cox proportional hazards models. For multivariable-adjusted analyses, the variables with *P* < 0.1 in the univariate analyses were considered as confounding factors. SAS version 9.4 (SAS Institute, Cary, NC) was applied for the data analysis, and a two-sided *P* <0.05 was considered significant.

## Results

### Characteristics of the study population

During the 5-year follow-up, 223 (4.98 %) new T2DM cases were identified. Among them, 85.65 % were male, 43.50 % were drinkers and 52.91 % were smokers. As the results shown in Table 1, the mean levels of BMI, systolic blood pressure, diastolic blood pressure, TG, TC and LDL-c in participants who developed T2DM were significantly higher than those who did not develop T2DM (all *P*<0.001). However, the baseline HDL-c concentration in new T2DM patients was significantly lower than that in participants who did not develop T2DM.

**Table 1:** Baseline characteristics of the study population

Variables	All participants	Diabetes	Non-diabetes	t/χ^2^	*P*
Age (years)	50.39±7.98	52.12±7.03	50.30±8.02	3.32	0.001
Male (%)	3125 (69.83)	191 (85.65)	2934 (69.00)	27.87	< 0.001
Body mass index (kg/m^2^)	24.05±2.61	25.54±2.51	23.97±2.59	8.82	< 0.001
Smoking (%)	1551 (34.66)	118 (52.91)	1433 (33.70)	34.54	< 0.001
Drinking (%)	1759 (39.31)	97 (43.50)	1662 (39.09)	1.73	0.189
Hypertension (%)	568 (12.69)	56 (25.11)	512 (12.04)	33.67	< 0.001
Antihypertensive drugs therapy (%)	720 (16.09)	54 (24.22)	666 (15.66)	11.48	0.001
Systolic blood pressure (mmHg)	115.41±12.81	121.17±13.07	115.11±12.72	6.93	< 0.001
Diastolic blood pressure (mmHg)	75.64±9.39	80.52±9.73	75.38±9.30	8.02	< 0.001
FPG (mmol/L)	5.33±0.44	5.91±0.47	5.30±0.42	20.99	< 0.001
TG (mmol/L)	1.67±1.26	2.26±2.02	1.64±1.20	7.20	< 0.001
TC (mmol/L)	4.92±0.85	5.14±0.97	4.91±0.84	3.92	< 0.001
LDL-c (mmol/L)	3.04±0.76	3.26±0.86	3.03±0.75	4.37	< 0.001
HDL-c (mmol/L)	1.35±0.34	1.20±0.28	1.35±0.34	6.52	< 0.001

### Mean, visit-to-visit variability and trajectory of TG and incident T2DM

Table 2 showed the influence of serum TG fluctuations on the subsequent probability of T2DM. Compared with the individuals in the normal range of TG levels (< 1.70 mmol/L), individuals with elevated TG concentrations (≥ 1.70 mmol/L) had a 1.67-fold (95 % CI: 1.25-2.21) higher risk of T2DM. Next, the mean TG level was further processed as a categorical variable (quartiles). Compared with persons in the lowest TG quartile (quartile 1, Q1), those with higher TG quartiles were significantly associated with subsequent T2DM probability in Q2 (HR: 3.38, 95 % CI: 1.63-7.00), Q3 (HR: 5.01, 95 % CI: 2.47-10.15) and Q4 (HR: 5.39, 95 % CI: 2.66-10.90). After excluding participants with prediabetes, the results also showed that multivariate-adjusted HRs (95 % CIs) for T2DM across the Q1, Q2, Q3 and Q4 of TG levels were 1.00, 3.58 (1.34-9.59), 5.02 (1.92-13.11) and 5.62 (2.13-14.81), respectively.

**Table 2:** Associations of mean, visit-to-visit variability and trajectory of TG with incident T2DM

TG	N (case)	Unadjusted HR (95 % CI)	*P*	Adjusted HR (95 % CI)	*P*
Mean level					
< 1.70 mmol/L	2962 (94)	1.00 (ref)		1.00 (ref)	
≥1.70 mmol/L	1513 (129)	2.79 (2.14-3.64)	< 0.001	1.67 (1.25-2.21)	0.000
Total					
Quartiles of the mean level (mmol/L)					
Q1 (0.32,0.97)	1106 (9)	1.00 (ref)		1.00 (ref)	
Q2 (0.98,1.37)	1146 (39)	3.98 (1.93-8.22)	0.000	3.38 (1.63-7.00)	0.001
Q3 (1.38,1.97)	1107 (77)	8.43 (4.22-16.81)	< 0.001	5.01 (2.47-10.15)	< 0.001
Q4 (1.98,12.14)	1116 (98)	10.83 (5.47-21.44)	< 0.001	5.39 (2.66-10.90)	< 0.001
*P*_*trend*_	< 0.001		< 0.001		< 0.001
Per 1-unit increase		1.41 (1.31-1.51)	< 0.001	1.26 (1.15-1.39)	< 0.001
Quartiles of SD					
Q1 (0.01,0.15)	1093 (27)	1.00 (ref)		1.00 (ref)	
Q2 (0.16,0.26)	1164 (42)	1.43 (0.88-2.32)	0.149	1.20 (0.73-1.95)	0.471
Q3 (0.27,0.43)	1078 (59)	2.28 (1.45-3.60)	0.000	1.60 (1.00-2.56)	0.049
Q4 (0.44,11.26)	1140 (95)	3.27 (2.13-5.02)	< 0.001	1.86 (1.20-2.89)	0.006
*P*_*trend*_	< 0.001		< 0.001		0.014
Trajectories					
Low	3263 (113)	1.00 (ref)		1.00 (ref)	
Moderate Moderate-high	980 (84) 200 (23)	2.49 (1.88-3.30) 3.82 (2.44-5.99)	< 0.001 < 0.001	1.51 (1.12-2.03) 2.55 (1.62-4.03)	0.007 < 0.001
High	32 (3)	4.34 (1.37-13.71)	0.012	1.93 (0.60-6.19)	0.268
*P*_*trend*_	< 0.001		< 0.001		0.000
Exclusion of prediabetes					
Quartiles of the mean level (mmol/L)					
Q1 (0.32,0.96)	1061 (5)	1.00 (ref)		1.00 (ref)	
Q2 (0.97,1.33)	1045 (21)	3.99 (1.51-10.59)	0.005	3.58 (1.34-9.59)	0.011
Q3 (1.34,1.93)	1047 (40)	7.93 (3.13-20.10)	< 0.001	5.02 (1.92-13.11)	0.001
Q4 (1.94,12.14)	1057 (51)	10.46 (4.18-26.22)	< 0.001	5.62 (2.13-14.81)	0.001
Trajectories					
Low	3056 (61)	1.00 (ref)		1.00 (ref)	
Moderate	940 (42)	2.30 (1.55-3.41)	< 0.001	1.36 (0.88-2.08)	0.163
Moderate-high	190 (12)	3.55 (1.91-6.59)	< 0.001	2.43 (1.28-4.63)	0.007
High	24 (2)	6.72 (1.64-27.58)	0.008	2.09 (0.48-9.03)	0.324

Adjusted confounders including age, gender, BMI, smoking, drinking, baseline TC level, baseline FPG level and antihypertensive drugs therapy

As the results shown in Table 2 and Supplementary Table 1, SD and CV of TG were processed as a categorical variable (quartiles). Multivariate-adjusted HRs (95 % CI) for T2DM across the Q1, Q2, Q3 and Q4 of TG/SD values were 1.00, 1.20 (0.73-1.95), 1.60 (1.00-2.56) and 1.86 (1.20-2.89), respectively (*P*_*trend*_=0.014). In addition, compared with individuals in the lowest quartile group, those in the highest quartile of TG/CV had a 49 % higher risk of T2DM (95 % CI: 1.05-2.12).

**Fig. 1:**
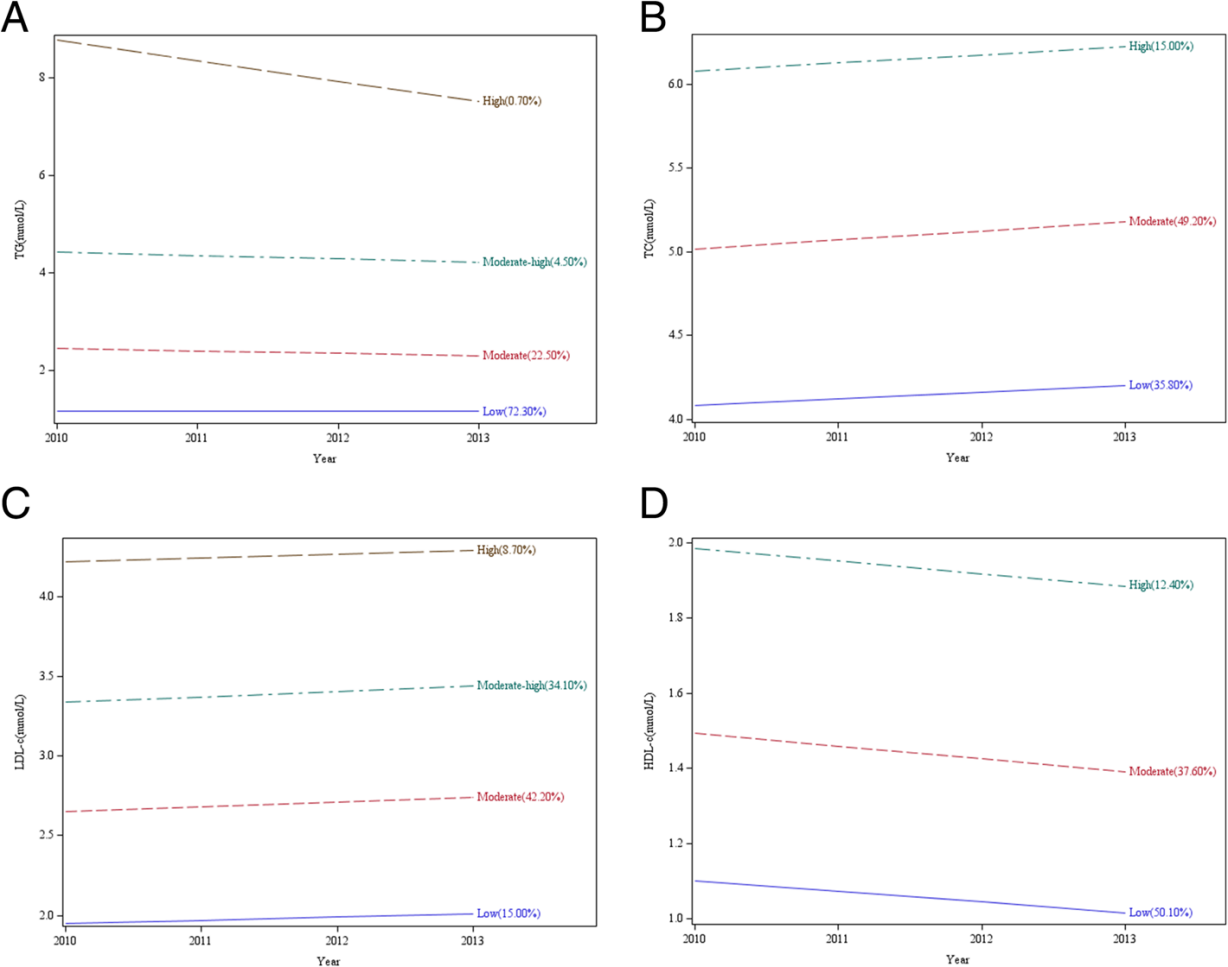
Change trajectories of serum TG, TC, HDL-c and LDL-c during 2010-2013. (**A**: Change trajectories of TG, **B**: Change trajectories of TC, **C**: Change trajectories of LDL-c and **D**: Change trajectories of HDL-c)

Four TG trajectory patterns were identified, which were named as “Low” (3263, 72.30 %), “Moderate” (980, 22.50 %), “Moderate-High” (200, 4.50 %) and “High” (32, 0.70 %), respectively (Fig. 1-A). Compared with the “Low” class, “Moderate” and “Moderate-High” classes were strongly associated with the subsequent T2DM risk, with adjusted HRs (95 % CIs) of 1.51 (1.12-2.03) and 2.55 (1.62-4.03), respectively (*P*_*trend*_ =0.000; Table 2). After excluding participants with prediabetes, the results showed that individuals with “Moderate-High” TG trajectory were still associated with subsequent T2DM risk compared with those with “Low” TG trajectory (HR: 2.43, 95 % CI: 1.28-4.63).

### Mean, visit-to-visit variability and trajectory of TC and incident T2DM

Table 3 showed the influence of TC fluctuations on the subsequent probability of T2DM. Compared with individuals in the normal range of TC level (< 5.18 mmol/L), those with elevated TC level (≥ 5.18 mmol/L) had a 35 % greater risk of T2DM (95 % CI: 1.04-1.77), however, the association was not significant in multivariate analysis. In addition, TC/mean, TC/SD and TC/CV did not show any influence on the future probability of T2DM (Table 3, Supplementary Table 2).

**Table 3:** Associations of mean, visit-to-visit variability and trajectory of TC with incident T2DM

TC	N (case)	Unadjusted HR (95 % CI)	*P*	Adjusted HR (95 % CI)	*P*
Mean level					
< 5.18 mmol/L	2915 (130)	1.00 (ref)		1.00 (ref)	
≥5.18 mmol/L	1560 (93)	1.35 (1.04-1.77)	0.026	1.01 (0.77-1.33)	0.926
Total					
Quartiles of the mean level (mmol/L)					
Q1 (2.48,4.38)	1114 (44)	1.00 (ref)		1.00 (ref)	
Q2 (4.39,4.88)	1126 (58)	1.32 (0.89-1.95)	0.166	1.07 (0.72-1.59)	0.738
Q3 (4.89,5.40)	1113 (51)	1.24 (0.83-1.85)	0.305	0.98 (0.65-1.48)	0.932
Q4 (5.41,8.74)	1122 (70)	1.63 (1.11-2.37)	0.012	1.07 (0.73-1.59)	0.724
*P*_*trend*_	0.082		0.085		0.955
Per 1-unit increase		1.26 (1.07-1.48)	0.006	1.01 (0.85-1.20)	0.884
Quartiles of SD					
Q1 (0.02,0.23)	1108 (47)	1.00 (ref)		1.00 (ref)	
Q2 (0.24,0.34)	1161 (52)	1.14 (0.77-1.68)	0.529	0.99 (0.66-1.47)	0.944
Q3 (0.35,0.47)	1103 (44)	1.04 (0.69-1.57)	0.847	0.96 (0.63-1.45)	0.834
Q4 (0.48,2.02)	1103 (80)	1.79 (1.25-2.56)	0.002	1.19 (0.83-1.72)	0.350
*P*_*trend*_	0.001		0.003		0.598
Trajectories					
Low	1608 (69)	1.00 (ref)		1.00 (ref)	
Moderate	2212 (108)	1.19 (0.88-1.61)	0.263	0.98 (0.72-1.33)	0.882
High	655 (46)	1.74 (1.20-2.53)	0.004	1.18 (0.80-1.74)	0.406
*P*_*trend*_	0.024		0.014		0.565
Exclusion of prediabetes					
Quartiles of the mean level (mmol/L)					
Q1 (2.48,4.37)	1044 (21)	1.00 (ref)		1.00 (ref)	
Q2 (4.38,4.87)	1057 (30)	1.41 (0.81-2.46)	0.230	1.34 (0.76-2.35)	0.315
Q3 (4.88,5.39)	1058 (22)	1.10 (0.60-2.00)	0.760	0.97 (0.53-1.78)	0.921
Q4 (5.40,8.74)	1051 (44)	2.12 (1.26-3.56)	0.005	1.56 (0.91-2.67)	0.109
Trajectories					
Low	749 (13)	1.00 (ref)		1.00 (ref)	
Moderate	1767 (46)	1.52 (0.82-2.81)	0.185	1.26 (0.68-2.36)	0.466
Moderate-high	1373 (43)	1.86 (1.00-3.47)	0.049	1.35 (0.71-2.56)	0.364
High	321 (15)	2.81 (1.34-5.90)	0.007	1.76 (0.82-3.79)	0.150

Adjusted confounders including age, gender, BMI, smoking, drinking, baseline TG level, baseline FPG level and antihypertensive drugs therapy

As the results shown in Fig. 1-B, three classes of TC trajectories including “Low” (1608, 35.80 %), “Moderate” (2212, 49.20 %) and “High” (655, 15.00 %), were identified in this study. However, the trajectories of TC did not have any influence on the future T2DM probability (Table 3).

### Mean, visit-to-visit variability and trajectory of LDL-c and incident T2DM

Table 4 showed the influence of LDL-c change characteristics on the subsequent risk of T2DM. Compared with individuals in the normal range of LDL-c concentrations (< 3.37 mmol/L), those with elevated mean LDL-c concentrations (≥ 3.37 mmol/L) were not associated with future T2DM probability (HR: 1.12, 95 % CI: 0.85-1.49). Although T2DM incidence increased with serum LDL-c level (*P*_*trend*_ =0.004), multivariable-adjusted analyses showed that higher LDL-c quartiles (Q2-Q4) were not associated with T2DM probability when compared with the lowest quartile (Q1). Additionally, neither SD nor CV of LDL-c was associated with future T2DM probability (Table 4 and Supplementary Table 3).

**Table 4:** Associations of mean, visit-to-visit variability and trajectory of LDL-c with incident T2DM

LDL-c	N (case)	Unadjusted HR (95 % CI)	*P*	Adjusted HR (95 % CI)	*P*
Mean level					
< 3.37 mmol/L	3354 (145)	1.00 (ref)		1.00 (ref)	
≥3.37 mmol/L	1121 (78)	1.54 (1.17-2.03)	0.002	1.12 (0.85-1.49)	0.426
Total					
Quartiles of the mean level (mmol/L)					
Q1 (0.00,2.49)	1125 (44)	1.00 (ref)		1.00 (ref)	
Q2 (2.50,2.92)	1109 (47)	1.16 (0.77-1.75)	0.477	1.02 (0.68-1.55)	0.920
Q3 (2.93,3.37)	1119 (54)	1.25 (0.84-1.87)	0.265	1.09 (0.73-1.63)	0.684
Q4 (3.38,6.57)	1122 (78)	1.75 (1.21-2.53)	0.003	1.16 (0.80-1.70)	0.432
*P*_*trend*_	0.004		0.015		0.848
Per 1-unit increase		1.30 (1.08-1.56)	0.005	1.02 (0.84-1.23)	0.859
Quartiles of SD					
Q1 (0.01,0.21)	1107 (39)	1.00 (ref)		1.00 (ref)	
Q2 (0.22,0.30)	1084 (50)	1.36 (0.90-2.07)	0.146	1.30 (0.85-1.98)	0.224
Q3 (0.31,0.42)	1195 (59)	1.41 (0.94-2.11)	0.098	1.09 (0.72-1.63)	0.688
Q4 (0.43,1.71)	1084 (75)	1.87 (1.27-2.75)	0.002	1.18 (0.80-1.76)	0.404
*P*_*trend*_	0.003		0.016		0.633
Trajectories					
Low	648 (30)	1.00 (ref)		1.00 (ref)	
Moderate	1915 (79)	0.95 (0.62-1.45)	0.808	0.78 (0.51-1.19)	0.250
Moderate-high	1527 (86)	1.23 (0.81-1.86)	0.337	0.91 (0.59-1.38)	0.642
High	3385 (28)	1.51 (0.90-2.53)	0.116	0.86 (0.51-1.45)	0.574
*P*_*trend*_	0.033		0.124		0.654
Exclusion of prediabetes					
Quartiles of the mean level (mmol/L)					
Q1 (0.00,2.49)	1064 (20)	1.00 (ref)		1.00 (ref)	
Q2 (2.50,2.92)	1057 (25)	1.34 (0.75-2.42)	0.328	1.15 (0.64-2.08)	0.647
Q3 (2.93,3.36)	1034 (26)	1.34 (0.75-2.39)	0.331	1.16 (0.65-2.09)	0.617
Q4 (3.37,6.57)	1055 (46)	2.28 (1.35-3.86)	0.002	1.63 (0.95-2.78)	0.074
Trajectories					
Low	609 (15)	1.00 (ref)		1.00 (ref)	
Moderate	1797 (36)	0.86 (0.47-1.57)	0.623	0.76 (0.41-1.39)	0.372
Moderate-high	1448 (48)	1.34 (0.75-2.39)	0.322	1.02 (0.57-1.84)	0.944
High	356 (18)	2.01 (1.01-3.99)	0.046	1.31 (0.65-2.62)	0.451

Adjusted confounders including age, gender, BMI, smoking, drinking, baseline TG level, baseline FPG level and antihypertensive drugs therapy

As shown in Fig. 1-C, four trajectories of LDL-c were classified, which were named as “Low” (648, 15.00 %), “Moderate” (1915, 42.20 %), “Moderate-High” (1527, 34.10 %) and “High” (3385, 8.70 %), respectively. However, nonsignificant association was detected between LDL-c trajectories and the incidence of T2DM.

### Mean, visit-to-visit variability and trajectory of HDL-c and incident T2DM

Table 5 showed the effects of serum HDL-c fluctuation on future T2DM probability. Although the incidence of T2DM was inversely related with HDL-c concentration (*P*_*trend*_ = 0.001), participants with decreased HDL-c levels (≤ 1.04 mmol/L) did not have a higher T2DM incidence when compared with those in the normal range (HDL-c > 1.04 mmol/L) (HR: 1.28, 95 % CI: 0.95-1.72). Next, the mean HDL-c level was further processed as a categorical variable (quartiles). Compared with the lowest quartile (Q1), the probability of T2DM was significantly reduced in the higher quartiles of HDL-c level, with adjusted HRs (95 % CI) of 0.55 (0.37-0.80) in Q3 and 0.38 (0.22-0.66) in Q4, respectively. After excluding participants with prediabetes, multivariate-adjusted HRs (95 % CI) for T2DM across quartiles of HDL-c levels (Q1, Q2, Q3 and Q4) were 1.00, 0.90 (0.57-1.41), 0.57 (0.33-0.97) and 0.35 (0.16-0.75), respectively.

**Table 5:** Associations of mean, visit-to-visit variability and trajectory of HDL-c with incident T2DM

HDL-c	N (case)	Unadjusted HR (95 % CI)	*P*	Adjusted HR (95 % CI)	*P*
Mean level					
>1.04 mmol/L	3532 (141)	1.00 (ref)		1.00 (ref)	
≤1.04 mmol/L	943 (82)	2.37 (1.80-3.11)	< 0.001	1.28 (0.95-1.72)	0.109
Total					
Quartiles of the mean level (mmol/L)					
Q1 (0.00,1.07)	1141 (98)	1.00 (ref)		1.00 (ref)	
Q2 (1.08,1.25)	1101 (65)	0.68 (0.49-0.93)	0.001	0.89 (0.65-1.24)	0.495
Q3 (1.26,1.50)	1125 (41)	0.37 (0.25-0.53)	< 0.001	0.55 (0.37-0.80)	0.002
Q4 (1.51,2.95)	1108 (19)	0.19 (0.11-0.31)	< 0.001	0.38 (0.22-0.66)	0.001
*P*_*trend*_	< 0.001		< 0.001		0.001
Per 1-unit increase		0.14 (0.09-0.23)	< 0.001	0.35 (0.20-0.63)	0.000
Quartiles of SD					
Q1 (0.01,0.07)	1056 (60)	1.00 (ref)		1.00 (ref)	
Q2 (0.08,0.10)	1012 (59)	0.93 (0.65-1.33)	0.675	0.92 (0.64-1.32)	0.650
Q3 (0.11,0.15)	1310 (60)	0.70 (0.49-1.01)	0.054	0.90 (0.63-1.30)	0.571
Q4 (0.16,0.52)	1092 (44)	0.56 (0.38-0.83)	0.004	0.78 (0.52-1.17)	0.234
*P*_*trend*_	0.160		0.014		0.699
Trajectories					
Low	2244 (164)	1.00 (ref)		1.00 (ref)	
Moderate	1680 (51)	0.38 (0.27-0.51)	< 0.001	0.52 (0.37-0.72)	< 0.001
High	551 (8)	0.18 (0.09-0.36)	< 0.001	0.38 (0.18-0.80)	0.011
*P*_*trend*_	< 0.001		< 0.001		< 0.001
Exclusion of prediabetes					
Quartiles of the mean level (mmol/L)					
Q1 (0.00,1.07)	1039 (50)	1.00 (ref)		1.00 (ref)	
Q2 (1.08,1.26)	1064 (34)	0.65 (0.42-1.01)	0.053	0.90 (0.57-1.41)	0.645
Q3 (1.27,1.51)	1077 (23)	0.39 (0.24-0.64)	0.000	0.57 (0.33-0.97)	0.040
Q4 (1.52,2.95)	1030 (10)	0.19 (0.09-0.37)	< 0.001	0.35 (0.16-0.75)	0.007
Trajectories					
Low	2100 (83)	1.00 (ref)		1.00 (ref)	
Moderate	1588 (29)	0.42 (0.28-0.64)	< 0.001	0.55 (0.35-0.88)	0.013
High	522 (5)	0.21 (0.09-0.53)	0.001	0.40 (0.15-1.04)	0.061

Adjusted confounders including age, gender, BMI, smoking, drinking, baseline TG level, baseline FPG level and antihypertensive drugs therapy

As shown in Fig. 1-D, three classes of HDL-c trajectories, named as “Low” (2244, 50.10 %), “Moderate” (1680, 37.60 %), and “High” (551, 12.40 %) were identified in this study. Compared with the “Low” HDL-c trajectory, “Moderate” and “High” trajectories could significantly reduce the incidence of T2DM during follow-up, with adjusted HRs (95 % CIs) of 0.52 (0.37-0.72) and 0.38 (0.18-0.80), respectively (*P*_*trend*_ <0.001; Table 5). After excluding participants with prediabetes, the “Moderate” HDL-c trajectory was still related to the reduced T2DM probability when compared with the “Low” trajectory (HR: 0.55, 95 % CI: 0.35-0.88). However, regardless of the exclusion of participants with prediabetes, SD and CV of HDL-c did not exhibit any association with future T2DM probability (Table 5 and Supplementary Table 4).

## Discussion

Recently, several studies have reported that fluctuations and changes in laboratory indices and biomarkers, such as hemoglobin, were significantly associated with long-term clinical status and disease prognosis [17, 18]. However, evidence for the effect of long-term serum lipid fluctuations on T2DM probability remains limited. This study simultaneously assessed the association of mean levels, visit-to-visit variabilities and trajectories of TG, TC, LDL-c, and HDL-c over 4 years with the subsequent 5-year probability of T2DM in Chinese adults. The following key findings were identified: participants with (1) larger TG/SD and “Moderate” and “Moderate-High” TG trajectories; and (2) lowest trajectories of HDL-c, were significantly associated with a greater risk of T2DM. Moreover, the associations of TG visit-to-visit variability, and change trajectories of TG and HDL-c with subsequent T2DM probability remained significant even after excluding the participants with prediabetes. These results strengthen the concept that long-term changes in different species of serum lipids should have different effects on future T2DM development.

Several lines of evidences supported the present findings [19–23]. For example, Taskinen et al. reported that appropriate 37.38 % of Swedish T2DM patients had increased serum TG concentrations [20]. In a Chinese study, Wang et al. suggested that blood TG level was independently associated with diabetes incidence among urban population [21]. Furthermore, Liu et al. reported that a one SD increase in serum levels of TG was associated with a 1.29-fold (1.10-1.52) higher risk of diabetes in participants with prediabetes [22]. In a Bangladeshi study, Bhowmik et al. also found that individuals with elevated TG levels had a 2.43-fold (1.46-4.04) greater risk of T2DM [23]. Herein, the results further reported that participants with long-term elevated TG levels, and higher mean TG concentrations had a higher risk of T2DM. Additionally, “Moderate”, “Moderate-High” and “High” TG trajectories could significantly increase the probability of T2DM. Therefore, the present findings further indicate that long-term TG level elevation should aggravate damages to the body, as reported previously [24–26].

Although previous researches have indicated that the chronic inflammatory state and non-enzymatic apolipoprotein glycation could contribute to the relative alteration of HDL-c composition and impairment of HDL-c function [27, 28], the precise influence of HDL-c on future T2DM probability remains unclear. For example, experimental studies have reported that insulin secretion was inversely correlated with HDL-c concentration, while insulin sensitivity was positively correlated with HDL-c concentration [29]. Consistent with the present findings, the results from a retrospective study found that increased trajectories of HDL-c were protective factors for T2DM compared with the decreased HDL-c trajectory [17]. However, in a comparative study, the results showed that HDL-c concentrations in T2DM patients did not have clear differences with those without diabetes [30]. Additionally, in a rural Chinese cohort study, Zhang et al. did not observe the significant association between elevated serum HDL-c concentrations and T2DM probability [31]. Thus, the long-term HDL-c fluctuation and its relationship with subsequent T2DM risk should be further investigated in future.

### Study strength and limitations

Strengths of this study include its longitudinal, prospective design and analysis of the change trajectory. However, this study had several limitations. First, some data, including diet, physical activity, lipid-lowering medicine, the type of antihypertensive drugs, education level and family income, were not collected, which could be important to the topic. Thus caution should be exercised when interpreting the present results. Second, although FPG and HbA1c examinations were conducted every year, the exact date of T2DM onset was not collected. Third, the study classified subjects into the most likely trajectory patterns, which could not account for the uncertainty of the classification. Fourth, this study was based on the population recruited from a single center in China, thus, sample representativeness might be limited. However, the dyslipidemia prevalence and other risk factors in the present participants is similar to those of the populations included in contemporary trials [32] and real-world registries from other countries and with various ethnicities [33]. These studies might partially support the generalizability of the present results. Finally, given that this study did not observe the influence of long-term TC and LDL-c fluctuations on future T2DM probability, more studies with large and diverse populations are needed to validate these findings.

## Conclusions

The present results suggest that long-term visit-to-visit variability of TG, and the change trajectories of TG and HDL-C were significantly associated with future T2DM probability. Moreover, these associations were not significantly affected after excluding participants with prediabetes. In clinical practice, these findings indicate that long-term TG and HDL-c fluctuations should be carefully measured in the population at high risk of T2DM. Moreover, there is a need to prioritize comprehensive lipid care for T2DM prevention.

## Supplementary information

**Additional file 1.**

## Data Availability

The datasets used and/or analyzed during the current study are available from the corresponding author on reasonable request.

## Abbreviations

BMI: Body mass index
FPG: Fasting plasma glucose
TG: Triglycerides
TC: Total cholesterol
LDL-c: Low-density lipoprotein cholesterol
HDL-c: High-density lipoprotein cholesterol
HbA1c: Glycosylated hemoglobin A1c
HRs: Hazard ratios
CIs: Confidence intervals
CV: Coefficient of variation
SD: Standard deviation
