# Supplementary material for: Associations of visit-to-visit variabilities and trajectories of serum lipids with the future probability of type 2 diabetes mellitus
Source: Lipids Health Dis. 2021 Nov 27;20:168. doi: 10.1186/s12944-021-01592-9 (PMC8627625; doi:10.1186/s12944-021-01592-9)
Supplement: Supplementary file 1 — Additional file 1. [file 12944_2021_1592_MOESM1_ESM.docx]

Supplementary Table 1. Associations of TG/CV of with the subsequent risk of T2DM

| Quartiles of TG/CV | N (case) | Unadjusted HR (95 % CI) | *P* | Adjusted HR (95 % CI) | *P* |
| --- | --- | --- | --- | --- | --- |
| Total |  |  |  |  |  |
| Q1 (0.02,0.14) | 1204 (54) | 1.00 (ref) |  | 1.00 (ref) | -- |
| Q2 (0.15,0.19) | 975 (47) | 1.09 (0.74-1.61) | 0.661 | 1.22 (0.82-1.81) | 0.331 |
| Q3 (0.20,0.27) | 1172 (43) | 0.79 (0.53-1.18) | 0.253 | 0.72 (0.48-1.08) | 0.117 |
| Q4 (0.28,1.28) | 1124 (79) | 1.53 (1.08-2.17) | 0.016 | 1.49 (1.05-2.12) | 0.027 |
| Exclusion of prediabetes |  |  |  |  |  |
| Q1 (0.02,0.13) | 967 (24) | 1.00 (ref) |  | 1.00 (ref) | -- |
| Q2 (0.14,0.19) | 1104 (29) | 1.02 (0.60-1.76) | 0.935 | 1.03 (0.60-1.77) | 0.922 |
| Q3 (0.20,0.27) | 1094 (22) | 0.77 (0.43-1.37) | 0.365 | 0.75 (0.42-1.34) | 0.336 |
| Q4 (0.28,1.28) | 1045 (42) | 1.55 (0.94-2.56) | 0.088 | 1.38 (0.83-2.28) | 0.216 |

Adjusted confounders including age, gender, BMI, smoking, drinking, baseline TC level, baseline FPG level and antihypertensive drugs therapy

Supplementary Table 2. Associations of TC/CV of with the subsequent risk of T2DM

| Quartiles of TC/CV | N (case) | Unadjusted HR (95 % CI) | *P* | Adjusted HR (95 % CI) | *P* |
| --- | --- | --- | --- | --- | --- |
| Total |  |  |  |  |  |
| Q1 (0.00,0.05) | 1188 (51) | 1.00 (ref) |  | 1.00 (ref) | -- |
| Q2 (0.06,0.07) | 1038 (52) | 1.24 (0.84-1.82) | 0.284 | 1.20 (0.82-1.78) | 0.349 |
| Q3 (0.08,0.10) | 1281 (55) | 1.11 (0.76-1.63) | 0.578 | 1.14 (0.78-1.67) | 0.514 |
| Q4 (0.11,0.40) | 968 (65) | 1.59 (1.10-2.30) | 0.013 | 1.16 (0.80-1.68) | 0.434 |
| Exclusion of prediabetes |  |  |  |  |  |
| Q1 (0.00,0.05) | 1119 (27) | 1.00 (ref) |  | 1.00 (ref) | -- |
| Q2 (0.06,0.07) | 978 (25) | 1.13 (0.65-1.94) | 0.670 | 1.22 (0.70-2.10) | 0.483 |
| Q3 (0.08,0.10) | 1218 (30) | 1.13 (0.70-1.89) | 0.656 | 1.21 (0.71-2.03) | 0.485 |
| Q4 (0.11,0.40) | 895 (35) | 1.68 (1.02-2.77) | 0.043 | 1.50 (0.91-2.49) | 0.114 |

Adjusted confounders including age, gender, BMI, smoking, drinking, baseline TG level, baseline FPG level and antihypertensive drugs therapy

Supplementary Table 3. Associations of LDL-c/CV of with the subsequent risk of T2DM

| Quartiles of LDL-c/CV | N (case) | Unadjusted HR (95 % CI) | *P* | Adjusted HR (95 % CI) | *P* |
| --- | --- | --- | --- | --- | --- |
| Total |  |  |  |  |  |
| Q1 (0.01,0.08) | 1294 (54) | 1.00 (ref) |  | 1.00 (ref) | -- |
| Q2 (0.09,0.11) | 1114 (55) | 1.14 (0.78-1.66) | 0.497 | 1.05 (0.72-1.53) | 0.821 |
| Q3 (0.12,0.14) | 875 (43) | 1.13 (0.76-1.69) | 0.538 | 0.99 (0.66-1.49) | 0.976 |
| Q4 (0.15,0.74) | 1187 (71) | 1.35 (0.95-1.92) | 0.096 | 0.99 (0.69-1.42) | 0.967 |
| Exclusion of prediabetes |  |  |  |  |  |
| Q1 (0.01,0.08) | 1224 (28) | 1.00 (ref) |  | 1.00 (ref) | -- |
| Q2 (0.09,0.11) | 1046 (30) | 1.18 (0.71-1.98) | 0.524 | 1.13 (0.67-1.89) | 0.647 |
| Q3 (0.12,0.14) | 829 (24) | 1.21 (0.70-2.09) | 0.488 | 1.12 (0.65-1.94) | 0.689 |
| Q4 (0.15,0.74) | 1106 (35) | 1.29 (0.79-2.13) | 0.309 | 1.05 (0.63-1.73) | 0.864 |

Adjusted confounders including age, gender, BMI, smoking, drinking, baseline TG level, baseline FPG level and antihypertensive drugs therapy

Supplementary Table 4. Associations of HDL-c/CV of with the subsequent risk of T2DM

| Quartiles of HDL-c/CV | N (case) | Unadjusted HR (95 % CI) | *P* | Adjusted HR (95 % CI) | *P* |
| --- | --- | --- | --- | --- | --- |
| Total |  |  |  |  |  |
| Q1 (0.00,0.06) | 1190 (54) | 1.00 (ref) |  | 1.00 (ref) | -- |
| Q2 (0.07,0.08) | 925 (48) | 1.09 (0.74-1.60) | 0.681 | 1.09 (0.74-1.63) | 0.656 |
| Q3 (0.09,0.11) | 1079 (52) | 0.91 (0.62-1.34) | 0.638 | 0.98 (0.67-1.44) | 0.906 |
| Q4 (0.12,0.38) | 1276 (69) | 0.97 (0.68-1.39) | 0.864 | 0.94 (0.65-1.35) | 0.722 |
| Exclusion of prediabetes |  |  |  |  |  |
| Q1 (0.00,0.06) | 1121 (27) | 1.00 (ref) |  | 1.00 (ref) | -- |
| Q2 (0.07,0.08) | 875 (25) | 1.12 (0.65-1.94) | 0.676 | 1.07 (0.62-1.85) | 0.807 |
| Q3 (0.09,0.11) | 1022 (29) | 1.01 (0.60-1.71) | 0.967 | 1.04 (0.62-1.76) | 0.879 |
| Q4 (0.12,0.38) | 1187 (36) | 1.04 (0.63-1.71) | 0.888 | 1.03 (0.62-1.69) | 0.924 |

Adjusted confounders including age, gender, BMI, smoking, drinking, baseline TG level, baseline FPG level and antihypertensive drugs therapy
